# ﻿A study of *Anthaxia* subgen. *Thailandia* Bílý, 1990 from China (Coleoptera, Buprestidae, Buprestinae)

**DOI:** 10.3897/zookeys.1154.97316

**Published:** 2023-03-21

**Authors:** Zhihao Qi, Hongmu Ai, Xueyou He, Rongxiang Su, Shouping Cai, Haitian Song

**Affiliations:** 1 Fujian Academy of Forestry, Fuzhou, Fujian, 350012, China Fujian Academy of Forestry Fujian China; 2 College of Plant Protection, Fujian Agriculture and Forestry University, Fuzhou, Fujian, 350002, China Fujian Agriculture and Forestry University Fujian China

**Keywords:** Guangxi, jewel beetle, new species, taxonomy, Yunnan

## Abstract

In this paper, the subgenus Thailandia Bílý, 1990 of the genus *Anthaxia* Eschscholtz, 1829 from China is reported, including two species: A. (T.) svatoplukbilyi Qi & Song, **sp. nov.** from Guangxi and A. (T.) rondoni Baudon, 1962 from Yunnan. The description and illustrations of the new species are provided, the illustrations and information of A. (T.) rondoni from Yunnan are given for the first time, and the diagnostic characters are provided to distinguish the new species from other related species.

## ﻿Introduction

Thailandia Bílý, 1990 is a small subgenus of the genus Anthaxia Eschscholtz, 1829 (Buprestinae, Anthaxiini). Bílý (1990) first established *Thailandia* as a monotypic genus of the tribe Anthaxiini Gory & Laporte, 1839 on account of the enlarged head, unusually short antennae, margined prosternal process, transverse elliptical scutellum, and particular shape of other structures, such as pronotum, anal sternite in females and elytral epipleura. In 2004, Bílý studied numerous related specimens from Southeast Asia and found that the characters mentioned in Bílý (1990) were insufficient for separating *Thailandia* as an independent genus. Then he downgraded *Thailandia* to the subgeneric rank in the genus *Anthaxia* (Bílý 2004). In a more recent study, *Thailandia* was well-defined due to the conspicuously wide head, convex frons, extremely wide vertex, and typical colouration (Bílý 2019).

Until now, only six species of the subgenus Thailandia have been described, and these are mainly distributed in Southeast Asia (Bílý 2005, 2019; Bellamy 2008). Recently, Bílý (2022) reported the occurrence of Anthaxia (Thailandia) rondoni Baudon, 1962 in Yunnan, China, for the first time but without more information. In this paper, we describe a new species, A. (T.) svatoplukbilyi Qi & Song, sp. nov., from Guangxi and give diagnostic notes. Additionally, we provide the first illustrations and further information on A. (T.) rondoni Baudon, 1962 from Yunnan.

## ﻿Materials and methods

### Measurement criteria are used as follows

Body length length between anterior margin of head and apex of elytra;

Body width the widest point across elytra;

Aedeagus length length between base and apex of parameres;

Aedeagus width the widest part of parameres.

### Abbreviations for collections in this study are

**FAF** Fujian Academy of Forestry, Fuzhou, China;

**KIZ**Kunming Institute of Zoology, Chinese Academy of Sciences, Kunming, China.

**Figure 1.**
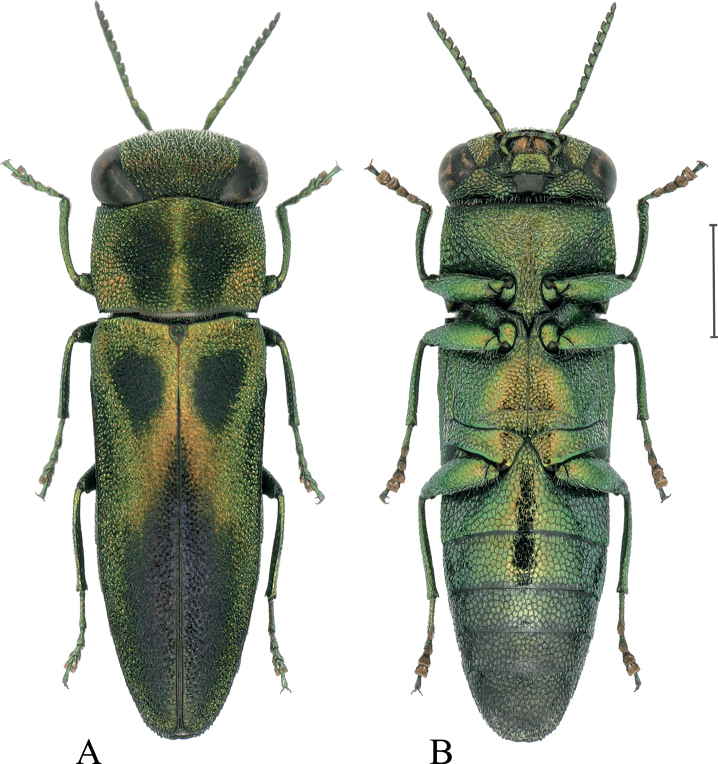
Habitus of Anthaxia (Thailandia) svatoplukbilyi Qi & Song, sp. nov. (holotype) **A** dorsal view **B** ventral view. Scale bar: 1 mm.

**Figure 2.**
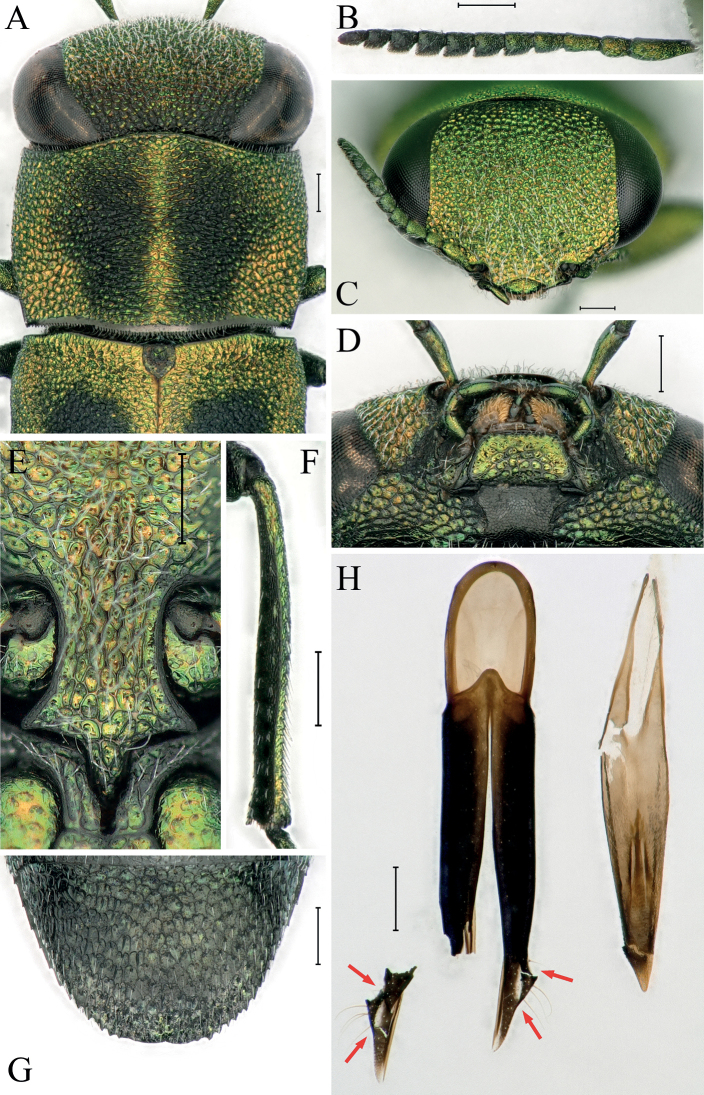
Detail features of Anthaxia (Thailandia) svatoplukbilyi Qi & Song, sp. nov. (holotype) **A** dorsal view of head, pronotum and scutellum **B** dorsal view of right antennae **C** frontal view of head **D** mentum **E** prosternal process **F** dorsal view of right metatibia **G** anal ventrite **H** dorsal view of aedeagus (left: parameres, right: medial lobe). Scale bars: 0.2 mm.

**Figure 3.**
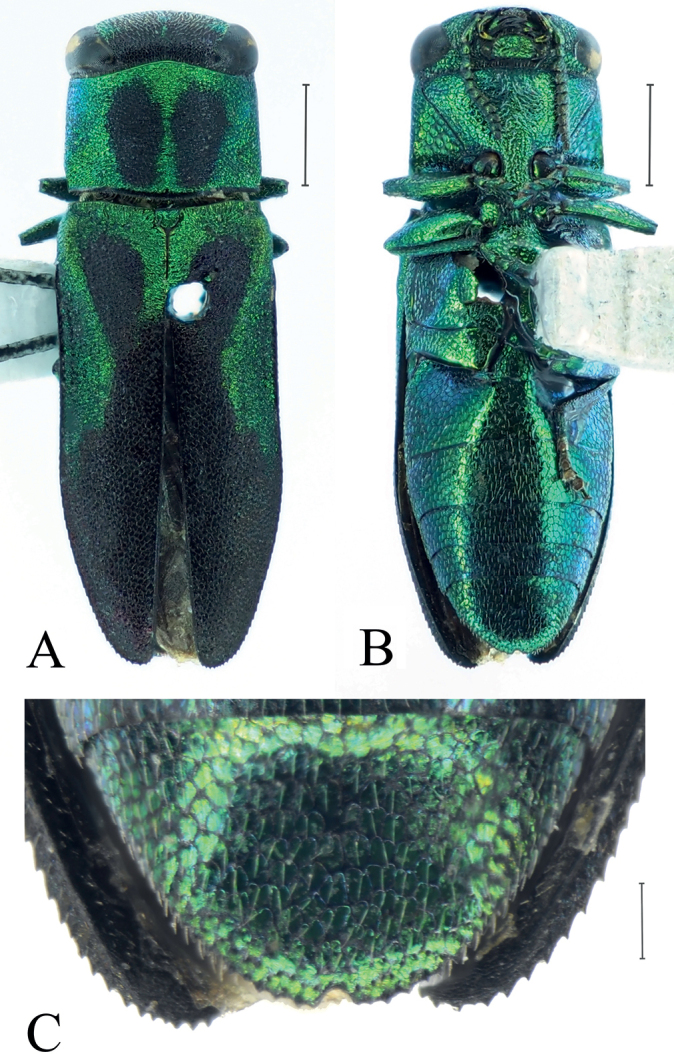
Female habitus of Anthaxia (Thailandia) rondoni Baudon, 1962 (Yunnan) **A** dorsal view **B** ventral view **C** anal ventrite. Scale bars: 1 mm (**A, B**), 0.2 mm (**C**).

Photographs (Figs 1, 2) of habitus and detail features were taken using a Keyence VHX-5000 digital microscope with a Keyence VH-Z20R zoom lens (20–200×). Photographs of Fig. 3 were taken using a Nikon D610 digital camera with a Nikon SMZ18 lens by Dr Kai-Qin Li (KIZ). The images were processed and combined into figures using Adobe Photoshop CC 2019.

## ﻿Taxonomy

### ﻿Family Buprestidae, Leach, 1815

**Subfamily Buprestinae, Leach, 1815**

**Tribe Anthaxiini Gory & Laporte, 1839**

**Genus *Anthaxia* Eschscholtz, 1829**

#### ﻿ Subgenus Thailandia

Taxon classificationAnimaliaColeopteraBuprestidae

Bílý, 1990

A2973CEE-8296-55EA-A4AC-833F6A292A21

##### Type species.

Anthaxia (Thailandia) paradoxa (Bílý, 1990).

#### ﻿ Anthaxia (Thailandia) svatoplukbilyi

Taxon classificationAnimaliaColeopteraBuprestidae

Qi & Song sp. nov.

4D783A30-B05E-5AB0-A01D-822A06A21E03

https://zoobank.org/FE47A429-B906-4BB6-8691-9BDA9D1A3516

Figs 1 , 2

##### Type locality.

China, Guangxi Zhuang Autonomous Region, Baise City, Leye County, Yachang Township, Ergou Mountain.

##### Type specimen.

***Holotype*** ♂ (FAF): China, Guangxi Zhuang Autonomous Region, Baise City [百色市], Leye County [乐业县], Yachang Township [雅长乡], Ergou Mountain [二沟], alt. 1200 m, 18.VII.2020, Ming-Biao Li leg.

##### Description of holotype.

Well preserved ♂ specimen. Length 5.41 mm, width 1.60 mm, length/width ratio: 3.4; aedeagus length: 1.55 mm, width: 0.31 mm, length/width ratio: 5.1.

***Body*** (Fig. 1A, B) small, elongate, spindle-shaped, whole body densely punctate; dorsal surface bicolour: light part metallic yellow to golden green and dark part metallic dark violet to black; most of ventral surface golden green, end of abdomen nearly black.

***Head*** (Fig. 2A–D) large, transverse, wider than anterior pronotal margin; most part golden green, posterior part nearly black. Frons (Fig. 2C) slightly convex, vertex 1.6× as wide as width of eye, with distinct white pubescence. Eyes large, ocular distance wide, lateral margins distinct convex, inner margins strongly concave at base. Sculpture of head homogeneous, consisting of small, dense, oval to polygonal cells. Antennae (Fig. 2B) compact and setose with 11 antennomeres, the length of antenna shorter than the total length of head and pronotum; 1^st^ antennomere longest, pear-shaped, 3.0× as long as wide, 2^nd^ antennomere subcylindrical, 1.7× as long as wide, 3^rd^ antennomere weakly triangular, 1.8× as long as wide, antennomeres 4–10 rhomboid, 1.1–1.5× as long as wide (not in the order of the antennomeres), terminal antennomere slightly ovoid, 1.9× as long as wide; antennomeres 1–6 in dorsal view golden green, antennomeres 7–11 in dorsal view black. Mentum (Fig. 2D) small, nearly trapezoidal, golden green. Maxillae yellowish brown, maxillar palpi golden green.

***Pronotum*** (Fig. 2A) short, 1.5× as wide as long, sculpture consisting of elongate, transverse, irregular cells in middle and rounded cells on both sides, cells without central grains; anterior margin curved, gently convex in the middle; posterior margin widely arcuate with a row of small, dense teeth; lateral margins slightly curved, weakly convergent near base; laterobasal pronotal depressions absent; pronotum golden green with two longitudinal, broad, distinct, black stripes nearly reaching both anterior and posterior pronotal margins, with an indistinct boundary between the two stripes (midline).

***Scutellum*** (Fig. 2A) small, subpentagonal, with several wrinkles and more finely microsculptured; the whole dark and slightly golden green.

***Elytra*** (Fig. 1A) elongate, spindle-shaped, 2.4× as long as wide, elytral sculpture almost homogeneous, consisting of fine, dense, simple punctures; lateral margins gradually narrowed from apical third and finely serrate, apex rounded. Anterior half of elytron mainly light (metallic yellow to golden green), with two large, basal, black spots; apical half of elytron mainly dark (dark violet to black), with wide, golden-green stripes along the lateral margins.

***Legs*** lustrous, with dense, short setae. Protibiae slightly curved inward; metatibiae (Fig. 2F) not modified, without teeth along inner margin.

***Ventral side*** (Fig. 1B) lustrous with dense, white setae; sternal part with ocellate sculpture; cells without central grains, each one with a white pubescence; prosternal process (Fig. 2E) widely expanded behind procoxae and sharply pointed apically (apical denticle), with two lateral dents at base of apex dent; lateral dents more obtuse than apical one. Abdominal ventrites with ocellate sculpture gradually smaller and darker apically; anal ventrite (Fig. 2G) lateral margins serrate, not notched at tip.

***Aedeagus*** (Fig. 2H) subparallel, apexes of parameres sharp and translucent, apical portion of each paramere lateral expand with one sharp outer tooth, base of the extension part translucent and completely surrounded by black chitinous parts (red arrows in Fig. 2H); medial lobe roughly serrate laterally, moderately acuminate apically.

##### Etymology.

This species is named in memory of the eminent Czech coleopterist, the late Dr Svatopluk Bílý, an excellent taxonomist in buprestid beetles. He helped us greatly and encouraged us in our work.

##### Distribution.

China (Guangxi).

##### Diagnosis.

In addition to the new species, the subgenus Thailandia contains six other species: A. (T.) capitata Kerremans, 1892, A. (T.) paradoxa (Bílý, 1990), A. (T.) phyllanthi Obenberger, 1956, A. (T.) rondoni Baudon, 1962, A. (T.) siamensis Bílý, 2005 and A. (T.) svobodai Bílý, 2005. Of these, A. (T.) rondoni is the one most similar to A. (T.) svatoplukbilyi Qi & Song, sp. nov. The following set of characters will help to recognize this new species more precisely from A. (T.) rondoni: 1) elytra 2.4× as long as wide, rather than 2.1–2.3×; 2) pronotum with two weakly limited black stripes nearly reaching both anterior and posterior pronotal margins (Fig. 2A), rather than with two small, black, slightly elongate, well-limited spots not reaching both anterior and posterior pronotal margins (Fig. 3A); 3) scutellum almost dark rather than metallic green or metallic blue; 4) base of apical extension part of each paramere with a small longitudinal translucent stripe completely surrounded by the black chitinous part (red arrow in Fig. 2H), rather than partly surrounded by the black chitinous part with an obvious gap near lateral tooth (see Bílý 2019: fig. 75). It is easier to separate the new species from the other related species: 1) bicoloured, rather than unicoloured as in A. (T.) paradoxa; 2) elytra with two large, nearly oval, basal, black spots, rather than without two large, basal, black spots as in A. (T.) capitata, A. (T.) paradoxa, A. (T.) phyllanthi, A. (T.) siamensis and A. (T.) svobodai; 3) metatibiae without teeth on inner margin, rather than with some teeth on inner margin as in A. (T.) capitata, A. (T.) phyllanthi and A. (T.) svobodai. Moreover, the combination of characters of the male genitalia of the new species differs from all other known species.

##### Remarks.

This new species is difficult to collect, and females, variation, and the host plant are all unknown.

#### ﻿ Anthaxia (Thailandia) rondoni

Taxon classificationAnimaliaColeopteraBuprestidae

Baudon, 1962

56BA2DD2-38A4-5661-BD57-F5ACCD223393

Fig. 3

Anthaxia rondoni Baudon, 1962: 28.
Anthaxia (Haplanthaxia) rondoni : Bílý 1997: 109.
Anthaxia (Thailandia) rondoni : Bílý 2004: 2.

##### Type localiy.

Central Laos, Tha Ngon.

##### Material examined.

1♀ (KIZ): China, Yunnan Province, Nujiang of the Lisu Autonomous Prefecture, Lushui City [泸水市], Liuku [六库], 11.VI.1983, Yong-Han Long leg.

##### Distribution.

China (Yunnan); Thailand; Laos; Vietnam; India (verification needed).

##### Remarks.

The colour and pattern of this species are variable. Body usually metallic blue or metallic green; elytra usually with two large, basal, black spots (see Baudon 1966: pl. 4 fig. b; Bílý 2005: fig. 3) or black part not separated by light part, forming a black Y-shape in the middle (Fig. 3A; Bílý 2019: fig. 18).

Bílý (2022) first reported the occurrence of A. (T.) rondoni from Yunnan, China. This is also the first record of the subgenus Thailandia in China. Unfortunately, neither figures nor collecting data of this species from China were given, while a specimen from “India” was provided in the colour figures. India was also a new record of this species but not mentioned in the catalogue’s distribution information. Thus, the distribution of A. (T.) rondoni in Yunnan and India is uncertain. Moreover, the distribution of the species in Vietnam (Bílý 2005) seems to be missing from the newest catalogue (Bílý 2022). In our study, the habitus (Fig. 3A, B) and anal ventrite (Fig. 3C) of a female A. (T.) rondoni from Yunnan are figured for the first time, and the collecting data confirms the occurrence of the species in Yunnan.

According to Bílý’s (2005) description, adults of this species have been repeatedly collected in northern and central Thailand and in southern Vietnam on the flowers of *Castanopsis* sp.

## ﻿Discussion

Our research proves again that the distribution of *Thailandia* is not limited in Southeast Asia, but that the subgenus also occurs in Yunnan, southwestern China. Moreover, we extend the distribution of this subgenus to Guangxi. As plants and climate are similar, we believe that *Thailandia* may also be present on Hainan Island and in southern Guangdong Province. In future research, we expect to have more specimens and discoveries from China and be able to better understand sexual individual differences, distribution, host plants, and life history of these interesting species.

## Supplementary Material

XML Treatment for Subgenus Thailandia

XML Treatment for Anthaxia (Thailandia) svatoplukbilyi

XML Treatment for Anthaxia (Thailandia) rondoni
